# Validation of energy intake from a web-based food recall for children and adolescents

**DOI:** 10.1371/journal.pone.0178921

**Published:** 2017-06-08

**Authors:** Anine Christine Medin, Bjørge Herman Hansen, Helene Astrup, Ulf Ekelund, Lene Frost Andersen

**Affiliations:** 1 Department of Nutrition, Institute of Basic Medical Sciences, Faculty of Medicine, University of Oslo, Oslo, Norway; 2 Department of Sport Medicine, Norwegian School of Sport Sciences, Oslo, Norway; McMaster University, CANADA

## Abstract

The purpose of this study was to validate estimated energy intake from a web-based food recall, designed for children and adolescents. We directly compared energy intake to estimates of total energy expenditure, calculated from accelerometer outputs, combined with data on weight and sex or resting energy expenditure prediction equations. Children (8–9 years) and adolescents (12–14 years) were recruited through schools in Norway in 2013 (N = 253). Results showed that more than one third (36–37%) were identified as under-reporters of energy. In contrast, only 2–4% were defined as over-reporters of energy. The mean energy intake was under-reported with -1.83 MJ/day for the entire study sample. Increased underestimation was observed for overweight and obese participants, the oldest age group (12–14 years), boys, those with parents/legal guardians with low educational level and those living in non-traditional families. In conclusion, energy intake from the web-based food recall is significantly underestimated compared with total energy expenditure, and should be used with caution in young people.

## Introduction

A healthy diet and normal body weight are key factors for preventing non-communicable diseases (NCDs), which are the leading causes of deaths worldwide [1]. Generally it takes time for NCDs to develop [2], and it is recognized that risk factors present in childhood may increase the risk of developing NCDs in adulthood [3]. Information regarding dietary exposure and energy intake in the first parts of humans’ lives is therefore of large interest in a public health perspective.

Dietary self-report methods such as food records, recalls and food frequency questionnaires have been widely used to assess total dietary or energy intake in children and adolescents [4], despite being prone to reporting bias [5]. Unfortunately, few alternatives exist, due to the fact that there are only a small number of recovery biomarkers available [6], observation of dietary intake for entire days are often not feasible [7], and the double-portion technique is both burdensome and expensive [8]. Thus, self-reported dietary methods need further refinement. New technology or altering and mixing elements from different methods have been suggested as a possible way forward [9]. Over the last years several new dietary assessment tools for children and adolescents have been developed [10]. Use of new technology (e.g. computers, mobile phones) provides clear advantages in terms of reduced data handling, and is preferred over paper-based methods among the young [11]. Yet, it is still not clear how the accuracy of dietary assessment is affected by using new technology [12], and more validation studies of new tools in the younger age groups are needed.

When validating a new dietary assessment tool, a comparison with an objective reference method, with non-correlated measurement errors, is preferred over a comparison with another dietary assessment method [13]. In weight stable individuals, there is a good agreement between energy intake (EI) and total energy expenditure (TEE) [14]. Hence, estimated EI can be evaluated against estimates of TEE [15]. Accelerometers measures physical activity fairly accurate and may be used to estimate TEE in combination with measured or estimated resting energy expenditure (REE) [16].

The aim of this study was to validate children’s and adolescents’ EI estimated from the web-based food recall (WebFR). This was done by a direct comparison of EI to estimates of TEE, calculated from accelerometer outputs combined with data on weight and sex or REE prediction equations. Furthermore, the proportion of acceptable- and non-acceptable reporters of EI was defined.

## Materials and methods

### Design

In this validation study of the WebFR, developed for use in a national dietary survey among 4^th^ and 8^th^ graders (8–9 and 12–14 years) in Norway, a total of 414 children, in these age groups, were invited through schools the fall of 2013 in the municipality of Bærum, Norway. Information regarding the study was provided orally and in writing to all children and their parents/legal guardians. Children were eligible for inclusion if they had Internet access at home and provided a valid email address to one of their parents / legal guardians. Two hundred seventy-six children got parental consent and were included, of which nine withdrew before study start or during the data collection. Participants used the WebFR concurrently with an ActiGraph GT3X+ accelerometer (ActiGraph LLC, Pensacola, FL, USA). Out of the 267 who completed the study, 14 had to be excluded due to incomplete data, of which 13 had less than two valid accelerometer recording days and one lacked entries in the WebFR. Thus, data from 253 (61.1% of all invited) was used in subsequent analyses.

### Ethics statement

Child assent and written parental consent were obtained from all participants. The Norwegian Data Protection Official for Research (NSD) approved the study (Project No. 32968). The study was conducted in accordance with the Declaration of Helsinki. A personal gift card containing two cinema tickets were given to all participants who completed the study.

### The WebFR

The web-based food recall (WebFR), described extensively in a previous paper [17], is a modified version of the Danish Web-based Dietary Assessment Software for Children (WebDASC) [18]. The interface includes an interactive character guiding the participants through each day’s eating occasions, in chronological order, using both audio and text in speech bubbles. To facilitate data entering, participants use a search function field or drop-down-lists with different categories containing around a total of 550 foods and beverages. A free text field is also available if the appropriate item does not exist among those listed. Pop-up elements are incorporated to remind the participants to enter in-between snacks, supplements, or other items often omitted from reports. All participants were instructed to enter everything they consumed for four consecutive days, including one weekend day retrospectively every evening at home after the last eating occasion. Parents/ legal guardians were instructed to assist the youngest participants (8–9 years).

### The accelerometers

The ActiGraph accelerometer is a small triaxial accelerometer used to provide objective measurements of physical activity and sedentary behaviors in free-living conditions. In this study all participants were instructed to wear the ActiGraph for seven consecutive days, including a weekend, and only to remove it during water activities (swimming, showering etc) and at night. They were given a demonstration on how to wear the ActiGraph on the right hip with an elastic band. In order to avoid recordings of any possible reactivity at startup, the participants were not informed that the accelerometers were programed to start the recordings the day after they started wearing them.

### Anthropometry

Height and body weight (TANITA TBF-300, Tanita Corporation, Tokyo, Japan) were measured, to the nearest 0.1kg and one millimeter, respectively, without shoes and in light clothing using standard procedures. To define overweight and obese participants, the age and sex-specific body mass index (ISO-BMI) from Cole et al. [19] was applied.

### Sex, age and family background

Information regarding sex and age, parental education level, parental ethnicity and family structure was provided from questions included in the written consent form completed by the parents/legal guardians.

### Data handling and statistics

Calculations in this paper were conducted using ActiLife (version 6.0, ActiGraph LLC, Pensacola, FL, USA), MS Excel (version 2010, Microsoft, Redmond, WA, USA) and IBM SPSS (version 22.0, 2013, IBM Corp, Armonk, NY, USA).

#### Accelerometer counts and individual physical activity level

Activity counts from the ActiGraph were used to calculate the rates of individual metabolic equivalents (METs) by using published algorithms (2005) [20]. The acceleration data was sampled at 30 Hz, and data from the vertical axis were used in the analyses. Non-wear periods were defined as periods of at least 20 minutes of consecutive zeroes. All activity between midnight and 6 am was excluded. Inclusion criteria were at least eight hours of recordings each day, for a minimum of two days. Individual physical activity levels (PALs) were expressed as average METS over 24-hour period time. For the non-valid accelerometer time, which included non-wear time (i.e. sleeping); a MET value of 1.2 was used.

#### Resting metabolic rate

Age, sex, weight and height specific equations from Henry [21] were used to estimate REE for each individual.

#### Prediction equations for total energy expenditure

The mean of the following three different equations were used to calculate TEE in MJ/day:

TEE1=((−SEX×380.9)+(CPM×1.177)+(WEIGHT(KG)×21.1)+706)×4.184÷1000

Equation from Ekelund et al. [22]. Boys = 0; Girls = 1. Activity counts are expressed in counts per minute (CPM).

TEE2=AEE+REE

AEE=((CPM×1.042)−(Sex×243.4)+238)×4.184÷1000

REE from Henry [21] and equation for activity energy expenditure (AEE) from Ekelund et al. [22]. Boys = 0; Girls = 1. Activity counts are expressed in counts per minute (CPM).

TEE3=REE×PAL

REE from Henry [21]. PAL expressed as average METS over 24-hour period time.

#### Estimated energy intake

Mean estimated EI from the WebFR recordings were calculated for the average of the four recording days, and for each of the four recording days. A one-way repeated measures ANOVA was conducted to compare EI across the recording days.

#### Pearson’s correlation between energy intake and total energy expenditure

Pearson’s correlations were calculated between EI and TEE for all participants and for subgroups of the sample.

#### Definition of acceptable-, under- and over-reporters of energy

Two different approaches were used in order to identify acceptable and non-acceptable reporters of energy intake. Participants were defined as either acceptable reporters (AR), under-reporters (UR), or over-reporters (OR). A theoretically impeccable reporter of energy intake, if weight stable, would fulfill the following:

1. EI/TEE = 1
2. EI/REE = PAL

However, such a perfect agreement cannot be anticipated. Thus, in the first approach, AR were defined as those within the 95% confidence limits (CL) of the agreement between reported EI and TEE, that takes into account the within-subject variation in reported EI and TEE in addition to the number of days of the dietary assessment method, as proposed by Black [15]. In this study, AR had EI/TEE from 0.72–1.28, UR had EI/TEE <0.72 and OR had EI/TEE >1.28. TEE is expressed as the mean of TEE1_,_ TEE2 and TEE3_,_ fully described earlier in this paper. Secondly, the well-established Goldberg cut-off approach [23] was used, in which AR were defined as those having a reported EI/REE within the 95% CL of agreement with their individual measured PAL, incorporating the within-subject variation in reported EI and REE, in addition to between-subject variation in PAL. That is, using the Goldberg cut-off approach, an AR with a PAL of e.g. 1.5 would have EI/REE between 1.07–2.11, UR would have EI/REE <1.07 and OR would have EI/REE>2.11. The within-subject coefficient of variation (CV) for reported EI was set to 23%, as suggested by Black [23]. A within-subject CV for TEE of 8.2%, based on doubly labelled water [24], was used when calculating the 95% CL for the EI/TEE agreement. For the Goldberg cut-offs, the standard CV for BMR of 8.5% was used to account for the variation in REE [23], in addition to a between-subject CV for PAL of 9.23% given from our own study-sample.

#### Bland-Altman plot

In order to explore and visualize if the agreement between EI and TEE differed across the mean scores of EI and TEE, a Bland-Altman plot was created.

#### Linear regression

Linear multiple regression analysis was used to investigate which variables contributed significantly to misreporting of EI, using ‘difference between EI and TEE (EI minus TEE)’ as the outcome. The variables ‘sex’, ‘age-group’, ‘weight status’, ‘parental educational level’, ‘parental ethnicity’ and ‘family structure’ were initially tested in univariate regression analysis. All were statistically significant at the 10% level, and were included in a multiple linear regression model. Subsequently, one variable, ‘parental ethnicity’, did not significantly contribute to the explained variance and was omitted from the model. No statistically significant interactions were found and all assumptions of normality and linearity were met.

#### Sensitivity analysis

Finally, a sensitivity analysis was conducted, to assess the validity of the reported EI after using a recommended approach to exclude implausible reporters of energy in nutrition epidemiology studies [25]; any individual with EI <2.09 MJ (500 kcal) or >14.64 MJ (3500 kcal) were excluded before running the previously described analysis.

## Results

The characteristics of the study sample are shown in Table 1. Forty-nine % of participants were 4^th^ graders (8–9 years) and 51% were 8^th^ graders (12–14 years). There were slightly fewer boys than girls, and 14% of all participants were either overweight or obese. The level of parental educational was high for most participants (77%); the majority had at least one parent/legal guardian with Norwegian ethnicity (86%), and lived in a traditional family (73%).

**Table 1:** Characteristics of study sample, measures of physical activity, reported energy intake, resting- and total energy expenditure, energy balance, and Pearson’s correlation between energy intake and total energy expenditure.

			Mean values							Correlations
		n (%)	CPM^1^ (SD)	PAL^2^ (SD)	REE^3^, MJ/day (SD)	EI^4^, MJ/day (SD)	TEE^5^, MJ/day (SD)	EI^4^/TEE^5^ (SD)	EI^4^-TEE^5^^,^ MJ/day (SD)	Pearson’s r (p-value)
All participants		**253** (100)	**616** (159)	**1.57** (0.15)	**5.49** (0.79)	**6.85** (2.13)	**8.69** (1.31)	**0.80** (0.26)	**-1.83** (2.31)	**0.16** (0.01)
Age					** **	** **	** **	** **	** **	
	4th graders (8–9 years)	**123** (49)	**664** (148)	**1.68** (0.11)	**4.99** (0.52)	**6.95** (2.02)	**8.32** (1.24)	**0.84** (0.24)	**-1.37** (2.03)	**0.31** (<0.001)
	8th graders (12–14 years)	**130** (51)	**571** (157)	**1.47** (0.09)	**5.97** (0.70)	**6.76** (2.23)	**9.04** (1.28)	**0.76** (0.27)	**-2.28** (2.48)	**0.08** (0.37)
Sex					** **	** **	** **	** **	** **	
	Girls	**139** (55)	**568** (138)	**1.54** (0.13)	**5.23** (0.66)	**6.37** (2.10)	**7.86** (0.93)	**0.82** (0.28)	**-1.49** (2.29)	**0.01** (0.97)
	Boys	**114** (45)	**675** (163)	**1.61** (0.15)	**5.82** (0.82)	**7.44** (2.03)	**9.69** (0.95)	**0.78** (0.22)	**-2.25** (2.28)	**-0.04** (0.65)
Iso-BMI cut off categories ^a^					** **	** **	** **	** **	** **	
	Normal weight	**218** (86)	**621** (160)	**1.58** (0.14)	**5.37** (0.68)	**7.07** (2.14)	**8.53** (1.23)	**0.84** (0.25)	**-1.47** (2.17)	**0.26** (<0.001)
	Overweight or obese	**35** (14)	**586** (150)	**1.56** (0.15)	**6.29** (0.96)	**5.52** (1.54)	**9.65** (1.41)	**0.58** (0.17)	**-4.13** (1.79)	**0.26** (0.13)
Parental education level ^b^					** **	** **	** **	** **	** **	
	Both parents/legal guardians low	**30** (12)	**571** (137)	**1.52** (0.11)	**5.78** (0.93)	**5.60** (1.95)	**8.80** (1.40)	**0.65** (0.22)	**-3.20** (2.30)	**0.09** (0.65)
	At least one parent/legal guardian high	**194** (77)	**623** (165)	**1.58** (0.15)	**5.42** (0.73)	**7.08 (**2.13)	**8.63** (1.26)	**0.83** (0.26)	**-1.55** (2.22)	**0.22** (<0.01)
	Missing	**29** (11)	**-**	**-**	**-**	**-**	**-**	**-**	**-**	**-**
Parental ethnicity					** **	** **	** **	** **	** **	
	At least one parent/legal guardian Norwegian	**217** (86)	**621** (163)	**1.58** (0.15)	**5.48** (0.77)	**6.98** (2.16)	**8.68** (1.30)	**0.81** (0.25)	**-1.71** (2.28)	**0.21** (<0.01)
	Both of other ethnic origin than Norwegian	**31** (12)	**568** (118)	**1.54** (0.13)	**5.56** (0.85)	**5.98 (**1.93)	**8.62 (**1.18)	**0.72** (0.28)	**-2.64** (2.51)	**-0.26** (0.16)
	Missing	**5** (2)	**-**	**-**	**-**	**-**	**-**	**-**	**-**	**-**
Family structure					** **	** **	** **	** **	** **	
	Traditional family ^c^	**185** (73)	**620** (161)	**1.58** (0.14)	**5.44** (0.72)	**7.08** (2.14)	**8.64** (1.26)	**0.83** (0.25)	**-1.56** (2.20)	**0.24** (<0.001)
	Other/non-traditional family	**60** (24)	**607** (161)	**1.56** (0.15)	**5.68** (0.91)	**6.28** (2.11)	**8.89** (1.41)	**0.72** (0.27)	**-2.61** (2.51)	**0.03** (0.82)
	Missing	**8** (3)	**-**	**-**	**-**	**-**	**-**	**-**	**-**	**-**

CPM, counts per minute; PAL, physical activity level; REE, resting energy expenditure; EI, energy intake; TEE, total energy expenditure; MJ, megajoule; SD, standard deviation.

^1^ Activity measured as counts/minute (CPM), from the accelerometer ActiGraph GT3X+.

^2^ Expressed as average metabolic equivalents (METs) over 24-hours, based on a minimum of eight hours of valid accelerometer time per day. A MET value of 1.2 was used for non-valid time.

^3^ REE from Henry's equation, based on sex, age, height and weight. Height and weight were measured.

^4^ EI calculated from dietary self-reports in a web-based food recall (WebFR).

^5^ TEE from the mean of three different prediction equations based on accelerometer counts, combined with data on weight and sex, or REE.

^a^ Based on the age and sex-specific body mass index (ISO-BMI) from Cole et al.

^b^ Low education defined as schooling limited up to high-school level at the most. High education defined as university-college or university level.

^c^ Family in which children are living with both their birth mother and biological father.

Table 1 shows that the participants had a mean PAL of 1.57. Moreover, they had a mean EI of 6.85 MJ/day, and the mean TEE was 8.67 MJ/day. The mean under-reporting of EI was -1.83 MJ/day for all participants, and -4.13 MJ/day among the overweight and obese. Pearson’s correlation between EI and TEE was 0.16 for the entire sample.

There was a significant difference in EI across the four recording days (Wilk’s Lamda gave p<0.001, and eta squared was 0.20). A steady increase in EI was observed from day one till four: 6.17 MJ, 6.47 MJ, 6.91 MJ and 7.84 MJ, respectively.

**Fig 1:**
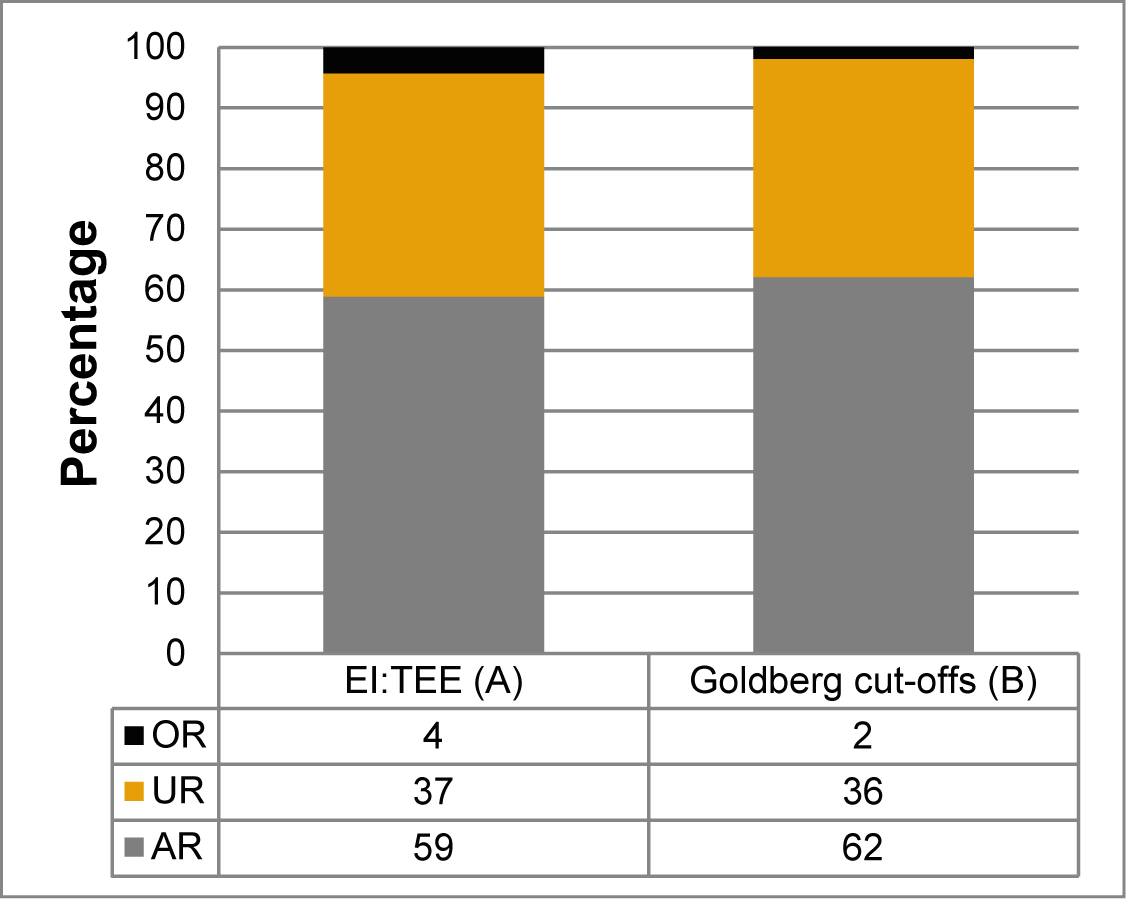
The percentage of AR, UR and OR, identified using two different approaches. AR, acceptable reporters; UR, under-reporters; OR, over-reporters; EI, energy intake; TEE, total energy expenditure. (A) AR were defined as those within the 95% confidence limits of the agreement between estimated EI from a web-based food recall (WebFR) and TEE calculated based on accelerometer counts, combined with data on weight, sex or REE. AR had EI:TEE from 0.72–1.28, UR had EI:TEE <0.72 and OR had EI:TEE >1.28. (B) The Goldberg cut-off approach was used, in which AR were defined as those having a reported EI:REE within the 95% CL of agreement of their individual physical activity level (PAL) measured by accelerometers. UR and OR were defined as those under and over this 95% CL, respectively.

Fig 1 shows that the proportion of AR varied between 59–62%, UR varied between 36–37% and OR varied between 2–4% when using two different calculation techniques. Thus, the differences between the two approaches are negligible.

**Fig 2:**
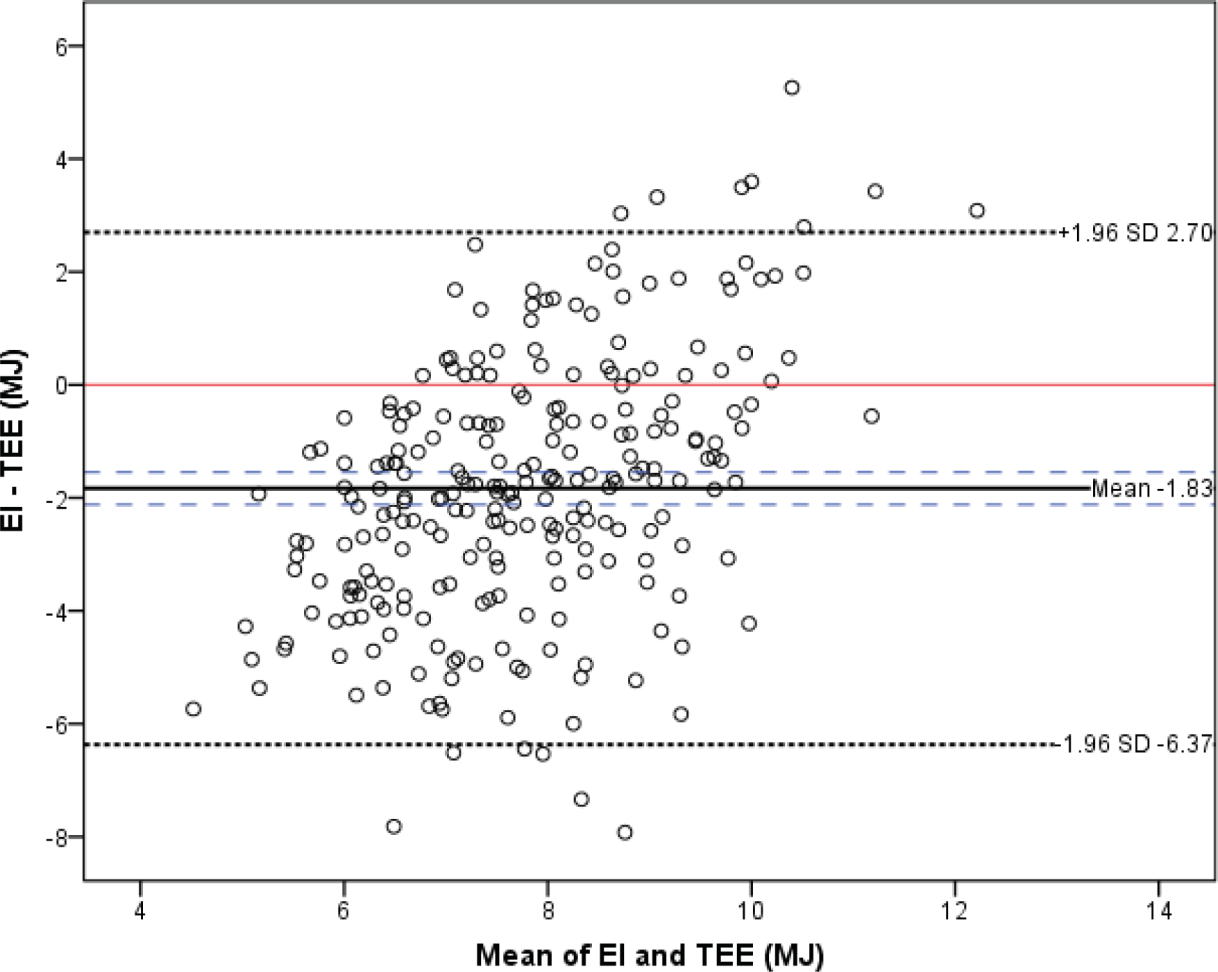
Bland-Altman plot displaying the difference between EI and TEE plotted against their mean. EI, energy intake; TEE, total energy expenditure; MJ, megajoule; SD, standard deviation. This visual plot demonstrates how the difference between estimated EI from a web-based food recall (WebFR) and TEE estimated based on accelerometer counts, combined with data on weight, sex or REE (Y-axis) varies with increasing levels on the scale (X-axis). The mean difference between EI and TEE is given by the solid thick line, together with the 95% CI for the mean, displayed in long stippled lines. The short stippled lines show +/- 1.96 SD of the mean difference between EI and TEE.

The Bland-Altman plot in Fig 2 gives a visual description of the difference between the reported EI from the WebFR and estimated TEE plotted against the mean of the two. The plot shows that the difference between EI and TEE deviate largely from 0, and more individuals are under-reporting, than over-reporting their energy intake. There is a tendency for more under-reporting at lower mean values of the two methods, and more over-reporting at higher mean values, suggesting possible bias.

A multiple linear regression model including variables associated with misreporting of EI is shown in Table 2. This model explains 24% of the variation in misreporting, defined by the difference between EI and TEE (EI minus TEE). BMI-category has the strongest impact: overweight or obese children under-reported their EI with -2.35 MJ/day more than the normal weight individuals. Moreover, increased under-reporting of EI was found for boys, the older children (12–14 years), those with parents/legal guardians with low educational level and those living in non-traditional families. These results are in line with the misreporting of EI in subgroups presented in Table 1.

**Table 2:** Variables associated with misreporting of reported energy intake, defined as the difference between EI and TEE (EI minus TEE).

Variables	Unadjusted (n = 224)		Adjusted (n = 224) ^a^	
	B, MJ/day	95% CI, MJ/day	B, MJ/day	95% CI, MJ/day
Sex ^b^	-0.66	(-1.26, -0.05)	-0.69	(-1.22, -0.16)
Age group ^c^	-0.71	(-1.31, -0.11)	-0.69	(-1.23, -0.16)
BMI-category ^d^	-2.55	(-3.36, -1.74)	-2.35	(-3.13, -1.58)
Parental education level ^e^	1.65	(0.79, 2.52)	1.17	(0.38, 1.96)
Family structure ^f^	-1.05	(-1.79, -0.30)	-0.90	(-1.56, -0.24)

EI, energy intake; TEE, total energy expenditure; B, beta coefficients; MJ, megajoule; CI, confidence interval.

^a^ Adjusted for all other variables in the model in a linear regression analyses.

^b^ Boys compared to girls (reference).

^c^ 12–14 year olds compared to 8–9 years olds (reference

^d^ Overweight or obese children compared to normal weight children (reference). Iso-BMI cut offs applied.

^e^ High parental education level compared to low education level (reference). A high level means that at least one parent/legal guardian has education at university or university college level.

^f^ 'Non-traditional family’ compared to ‘Traditional family’ in which children are living with both their birth mother and biological father (reference).

Sensitivity analyses showed that the overall results were not affected, as only one participant was excluded using the recommended cut-offs.

## Discussion

### Main findings

More than one third of all participants (36–37%) were identified as under-reporters (UR) in this study, when comparing estimated EI from a web-based food recall (WebFR) to TEE calculated from objective accelerometer counts, combined with data on weight, sex or REE. In contrast, only 2–4% were defined as over-reporters (OR). The mean under-reporting of EI was -1.83 MJ/day for the entire study sample. Increased underestimation was observed for overweight and obese participants, the oldest age group (12–14 years), boys, those with parents/legal guardians with low educational level and those living in non-traditional families.

### Comparisons with previous work

EI estimated from the Danish WebDASC system has previously been evaluated against TEE estimated from accelerometer counts and data on age, sex, height and weight [26]. Due to the similarities between instruments, we expected similar results. However, data from the Danish WebDASC suggested approximately 20% under-reporters and 20% over-reporters (Biltoft-Jensen et al.), compared with 36–37% UR, and 2–4% OR in this study. Moreover, the mean reported EI and the estimated TEE in the Biltoft-Jensen study [26] were not significantly different with a mean under-reporting of only -0.04 MJ/day, compared to our under-reporting of -1.83 MJ/day. About 50% of our participants were adolescents, who are known to be more influenced by social desirability that reduces their reporting accuracy [27], whereas the age range in Biltoft-Jensen et al.’s study was 8–11 years [26]; this may partly explain differences between studies. Besides, we had a higher proportion of overweight and obese individuals, in addition to individuals with a diverse ethnic background, compared with Biltoft-Jensen et al. [26], who described their study population as relatively homogeneous in respect of ethnic, social and cultural background [28].

Results from other studies, in which accelerometers have been used to validate estimated EI from more traditional paper-based methods among children, are in line with our study: suggesting the proportion of under-reporters is a large problem [29–31]. For example, estimated EI using a paper-based pre-coded food diary for four days in nine year old children suggested under-reporting of -1.8 MJ/day [30]. This is similar to our observations among the eight-nine year olds showing under-reporting of -1.4 MJ/day. Moreover, underestimation was larger in boys compared with girls [30], this is also in line with findings from the present study. Severe under-reporting was also observed in a similar validation study of a paper-based pre-coded food diary among 13 year olds; the mean difference between EI and TEE showed under-reporting that varied from -1.3 to -4.8 MJ/day [29]. Rothausen et al. report a difference between EI from a seven days food diary and TEE of -2.7 and -2.1 MJ/day for 12–13 year old boys and girls, respectively [31], which is comparable to our observations. However, these authors report better reporting accuracy for the same individuals using 2 x 24 hour recalls, and in seven to eight year old children. The effect of sex on misreporting of EI seems inconsistent in the literature, also when using the gold standard doubly labeled water as the reference [5]. In summary, our results corroborate previous observations using the traditional paper-based methods. A possible explanation for this may be that the inherent challenges with the dietary assessment methodology is not necessarily bypassed by the new technology alone, as suggested by Illner et al. [12].

Low reported intakes have been observed in the last days of long recording periods (>4 days) in adults [32, 33], explained by participant fatigue. We observed higher reported EI during the last recording days; hence, it is unlikely that participant fatigue have contributed to the under-reporting in this study. We speculate if the observed increase in EI over the recording days was caused by a learning effect, or if it reflects the day-of-the-week variation between recording days (day 3 and 4 held all Fridays and Saturdays, respectively). Supportive of the latter, Lillegaard et al. reported significantly higher EI Fridays and Saturdays, compared to week-days, in 9-year old children [34].

Under-reporting was greater in magnitude in overweight and obese children. This finding was expected, and has been reported previously, for example in a review by Burrows et al. in which doubly labelled water was used as the reference method [5]. Svensson et al. found under-reporting of EI of -2.84 MJ/day, when they assessed EI using a food record combined with digital cameras, and compared it to estimated TEE based on accelerometer counts and data on temperature, weight, height, sex, and age, among overweight and obese 8–12 year olds [35]. The under-reporting of EI was -4.1 MJ/day in our sample of overweight and obese participants. It is likely that the participants’ young age may have been contributing to higher reporting accuracy in the study of Svensson et al., as children’s dietary reporting is less biased before entering adolescence [36]. Additionally, the innovative element of using digital cameras in addition to the food record, may have improved the reporting accuracy. This is supported by a review paper on image-assisted dietary assessment among adults [37], in which the use of digital images as the primary record or in addition to traditional methods reduced underreporting. Methodologically new dietary assessment methods in the younger age groups have also been developed [10]. An example of a tool being more than just technologically new is the Technology Assisted Dietary Assessment (TADA) food record application, in which users take images of foods and beverages at all eating occasions using a mobile device [38]. However, no validation studies of the TADA, or similar methods, using objective reference methods in children or youth have been published, to our knowledge.

### Strengths

The use of accelerometers is a strength in this study, as accelerometers has demonstrated to be objective, accurate and reliable tools to measure physical activity, and is also considered as a preferred choice when estimating energy expenditure [16]. Moreover, the ActiGraph, which was used in this study, is the most commonly used accelerometer in physical activity research for children and adolescents [16].

All participants in this study were instructed to wear the accelerometer for seven consecutive days, and the sample achieved a mean of five valid measurement days. This strengthens the estimates of the individually estimated TEE and PAL. Further, in order to reduce the challenge with reactivity, the first day of wearing the accelerometer was omitted, that is, the accelerometers were programmed to start the day after the participants started wearing them.

We estimated TEE as the mean of three different algorithms, based on the statistical principle ‘wisdom of select crowds’, which postulates that averaging a small number of selected estimates based on expertise, will often outperform a single estimate taken as the best [39]. Moreover, aggregate prediction equations have shown to perform better than single prediction equations, when predicting body composition and resting metabolic rate [40]. The rationale is that by averaging, errors randomly distributed between uncorrelated estimates will cancel each other out [39, 40]. Consistent with this, our use of two different approaches to assess the proportion of AR, UR and OR, also ensure more robust data.

### Limitations

The equations from Ekelund et al. [22], used to estimate TEE and AEE from accelerometer counts and data on weight and sex, were developed in nine year old children. Therefore these equations may be less accurate for the 8^th^ graders (12–14 years) included in this study.

Moreover, although accelerometers have the advantage that they can assess physical activity objectively, and thus estimate AEE and TEE, if combined with data on weight, sex or REE, there are also several well-known limitations to their ability to assess AEE and thus TEE at the individual level [16].

Because participants were only weighed once in this study, we cannot discriminate between those who were truly in negative or positive energy balance, and thus under eating or over eating compared to their true energy needs, and those who were in energy balance and simply misreported their EI.

### External validity

The activity counts in our study were 664 and 571 CPM for 4^th^ graders (8–9 years) and 8^th^ graders (12–14 years), respectively. In comparison, a nationally representative Norwegian physical activity survey [41] observed a mean CMP of 653 for nine year olds, 421 CPM for 15 year old girls and 494 CPM for 15 year old boys. Given the age-related decline in physical activity level [42], the physical activity level of our sample is similar to the nationally representative data. Moreover, parental educational level, ethnic background and weight status in our participants are comparable to the population from urban and semi-urban areas in Norway, demonstrated in a previous paper covering the same study sample [43]. Therefore the results in this paper can probably be extrapolated to children and adolescents living in similar areas in Norway.

## Conclusions

The WebFR significantly underestimated EI compared with TEE estimated from accelerometer counts, combined with data on weight, sex or REE, in children (8–9 years) and adolescents (12–14 years). The magnitude of underestimation was influenced by overweight and obesity, sex, age, parental education level and family structure. The level of under-reporting of energy is in line with traditional paper based methods, and the estimated EI from the WebFR should be used with caution, equally as estimated EI from traditional dietary self-reports.
